# Abdominal massage modulates gut microbiota and brain-gut peptides in insomnia model rats

**DOI:** 10.3389/fmicb.2025.1720248

**Published:** 2025-12-02

**Authors:** Junchang Liu, Gulaisaer Aikebaier, Xusheng Lu, Xingping Zhang

**Affiliations:** 1 The Fourth Clinical Medical College of Xinjiang Medical University, Ürümqi, China; 2 College of Traditional Chinese Medicine of Xinjiang Medical University, Ürümqi, China; 3 Hospital of Traditional Chinese Medicine Affiliated to Xinjiang Medical University, Ürümqi, China; 4 Xinjiang Uygur Autonomous Region Institute of Traditional Chinese Medicine, Ürümqi, China

**Keywords:** abdominal massage, insomnia, microbiota-gut-brain axis, inflammatory factors, brain-gut peptides

## Abstract

**Background:**

Abdominal massage is a therapeutic intervention in traditional Chinese medicine for managing insomnia; however, its underlying mechanisms remain incompletely understood. This study aimed to investigate the effects of abdominal massage on the gut microbiota and brain-gut peptides in a rat model of insomnia from the perspective of the microbiota–gut–brain axis.

**Methods:**

Forty-eight male Wistar rats were randomized into control, model, abdominal massage (Abd massage), and zolpidem groups (*n* = 12). An insomnia model was induced by intraperitoneal injection of 4-chloro-DL-phenylalanine (PCPA). The Abd massage group and the zolpidem group, respectively, received 14 days of abdominal massage and zolpidem treatment. Hippocampal histopathology was evaluated with hematoxylin and eosin (HE) staining. Serum levels of interleukin-1 beta (IL-1β), interleukin-6 (IL-6), tumor necrosis factor-alpha (TNF-*α*), vasoactive intestinal peptide (VIP), growth hormone (GH), substance P (SP), and cholecystokinin-8 (CCK8) were measured by enzyme-linked immunosorbent assay (ELISA). The gut microbiota composition was examined using 16S rRNA sequencing.

**Results:**

Behavioral experiments in a rat model of insomnia demonstrated that abdominal massage significantly extended sleep duration. The treatment alleviated histopathological damage in the hippocampus and regulated brain-gut peptide levels in both colon and brain tissues. Additionally, abdominal massage modulated gut microbiota structure, reducing the relative abundance of *Bacteroidetes* and *Proteobacteria* and increasing that of *Firmicutes*, *Lachnospiraceae_NK4A136_group*, *Clostridia*, and *Clostridiales*. Spearman correlation analysis revealed significant associations between microbial abundance and biochemical indicators. PICRUSt2 analysis further implicated carbohydrate metabolism, amino acid metabolism, and transcriptional regulation in the pathogenesis of insomnia.

**Conclusion:**

The results of this study demonstrate that abdominal massage ameliorates insomnia and increases sleep duration. This effect is associated with the regulation of brain-gut peptide levels and the restoration of gut microbiota diversity and structure. These findings suggest that the microbiota-gut-brain axis may be involved in the therapeutic mechanism of abdominal massage for insomnia.

## 1 Introduction

Insomnia, characterized by persistent difficulties in sleep initiation, maintenance, or early awakening, is by far the most common sleep disorder and the second most common neuropsychiatric disorder (Van Someren, 2021). It poses significant risks to physical and mental health, including cardiovascular diseases, metabolic dysregulation, and cognitive impairment (Perlis et al., 2022). Between 10 and 20% of people have insomnia, and 50% of those cases progress chronically (Buysse, 2013). Current management relies on Cognitive-Behavioral Therapy for Insomnia (CBT-I) and pharmacotherapy; however, clinical implementation faces challenges, including CBT-I practitioner shortages and medication-related adverse effects, underscoring the need for non-pharmacological therapies targeting sleep regulation mechanisms (Buysse, 2013; Rossman, 2019; Krystal et al., 2021).

Traditional Chinese medicine (TCM) massage therapy has been widely used in clinics as a form of external treatment. Abdominal massage is a non-invasive, gentle massaging of the abdomen to treat various conditions, such as insomnia, type 2 diabetes, fatigue syndrome, and other medical problems (Li et al., 2017; Qi et al., 2021; Wang et al., 2022; Xie et al., 2022). TCM theory holds that the abdomen is the root of all diseases. Modern neuroanatomy recognizes its dense enteric neuronal networks—termed the “second brain” (Avetisyan et al., 2015). Emerging evidence suggests that abdominal massage may rebalance hypothalamic–pituitary–adrenal axis activity, potentially restoring sleep architecture by modulating the gut-brain axis (GBA) (Zhang et al., 2021). Despite these advances, the precise mechanisms by which abdominal massage alleviates insomnia remain unexplored.

The GBA constitutes a bidirectional communication network integrating the gastrointestinal tract and the Central Nervous System (CNS) through coordinated neural, immune, and endocrine pathways. This framework has expanded to encompass the microbiome, forming the Microbiota-Gut-Brain Axis (MGBA), which mediates bidirectional interactions between gut microbes and sleep regulation (Cryan and O'Mahony, 2011; Mayer, 2011; Li et al., 2018). Gut microbiota modulate sleep by producing neuropeptides and metabolites, while sleep deprivation disrupts microbial composition and metabolic activity, inducing dysbiosis (Aresti Sanz and El Aidy, 2019; Wang et al., 2024). Liu et al. reported significant differences in gut microbial diversity and composition between 10 patients with chronic insomnia and 10 matched healthy controls (Liu et al., 2019). Similarly, Li et al. observed reduced microbial diversity in patients with both acute and chronic insomnia, with more pronounced reductions in the chronic group (Li et al., 2020). Both studies suggested that an increased relative abundance of certain bacterial phyla may serve as potential biomarkers for insomnia. It is now established that numerous neuropeptides are co-produced by central and peripheral neurons, as well as by endocrine cells within the gastrointestinal tract and other organs (Holzer and Farzi, 2014). These neuropeptides function as dual-purpose mediators, acting as both hormones and neurotransmitters to coordinate brain–gut signaling (Aresti Sanz and El Aidy, 2019). Consequently, the GBA represents a promising therapeutic target for novel treatment strategies. This concept is central to a growing paradigm in which various therapeutic modalities, including TCM, are being explored for their ability to rebalance the microbiota-gut-brain axis to treat insomnia (Feng et al., 2023).

In this study, we investigated the therapeutic effects of abdominal massage in a 4-chloro-DL-phenylalanine (PCPA)-induced insomnia rat model, focusing on its modulation of the brain-gut axis. Our findings demonstrate that both abdominal massage and zolpidem ameliorate insomnia symptoms by restoring microbiota diversity and composition disrupted by PCPA modeling and enriching beneficial microbial taxa. These results reveal that abdominal massage represents a potential therapeutic strategy for insomnia.

## 2 Methods

### 2.1 Animals and PCPA-pretreated insomnia rat model

Forty-eight male Wistar rats (6–8 weeks, 180–220 g) were obtained from the Laboratory Animal Center of Xinjiang Medical University (Laboratory Animal Production License No SYXK[Xin]2018–0003). Animals were maintained under specific pathogen-free (SPF) conditions with controlled environmental parameters: ambient temperature 23 ± 2 °C, relative humidity 40–60%, and 12:12 h light–dark cycle. Food and water were provided ad libitum throughout the study. Following a 7-day acclimatization period, rats were randomly allocated into either the control group (*n* = 12) or the PCPA group (*n* = 36). PCPA group animals received daily intraperitoneal injections of PCPA (300 mg/kg in 0.9% saline, HY-B1368, Med Chem Express, Monmouth Junction, NJ, USA) for three consecutive days, while control animals received equivalent volumes of saline vehicle.

Successful model establishment was confirmed through behavioral validation to secondary randomization of PCPA-treated rats into three groups (*n* = 12 per group): model, abdominal (Abd) massage, and Zolpidem. All experimental procedures were approved by the Institutional Animal Care and Use Committee of Xinjiang Medical University (IACUC Approval No. 20190226-09). All personnel involved in animal handling completed the mandatory ethics training certification.

### 2.2 Zolpidem treatment

Rats in the zolpidem group received daily intragastric administration of zolpidem solution (0.92 mg/kg, Sanofi, Hangzhou, China) for 14 consecutive days, while the non-drug groups were administered an equivalent volume of saline following identical protocols.

### 2.3 Abdominal massage treatment

Rats in the Abd massage group were secured in custom-designed restraining devices with abdominal exposure. The therapeutic manipulation was centered on Shenque acupoint (CV8), administered once daily for 14 consecutive days with the following two sequential phases: (1) clockwise circular friction (concentric with 25 mm diameter) applied with the experimenter’s thumb at 4 N pressure (100 cycles /min) for 5 min, (2) equivalent counterclockwise manipulation for 5 min. The direction was anatomically defined: clockwise motion proceeded from the cranial to the right lateral, caudal, and left lateral aspects of the abdomen; counterclockwise motion was the exact inverse. All procedures were performed by two certified practitioners who underwent standardized training. To ensure consistency across all practitioners and sessions, the pressure magnitude and application frequency were continuously monitored and recorded in real-time using a FingerTPS II wireless pressure measurement system (Pressure Profile Systems, Los Angeles, USA). Any deviation from the preset parameters was immediately corrected during the intervention.

### 2.4 Pentobarbital-induced sleep test

After 30 min of the last intervention, all rats received an intraperitoneal injection of pentobarbital sodium (35 mg/kg, F20041117, SCRC, Shanghai, China) for sleep induction. Sleep parameters were quantified using two primary endpoints: sleep latency (time from injection to persistent loss of righting reflex >60 s) and total sleep duration (time between righting reflex disappearance and spontaneous recovery). Under controlled environmental conditions, all assessments were conducted by observers blinded to the group assignments.

### 2.5 Tissue sampling

Following the final experimental intervention, rats were individually housed in sterilized metabolic cages for fecal specimen collection to ensure sample integrity and prevent cross-contamination. Fecal pellets were immediately flash-frozen in liquid nitrogen. Animals subsequently underwent overnight fasting with ad libitum access to water. Anesthetized via intraperitoneal injection of pentobarbital sodium (40 mg/kg), blood collection via abdominal aortic puncture. Blood samples were centrifuged at 3000 × g for 10 min at 4 °C to isolate serum, which was aliquoted and stored at −80 °C. Tissues were dissected within 3 min post-mortem: whole brains and colons were excised, with hypothalamic, hippocampal, and brainstem regions isolated on an ice-cold dissection tray before snap-freezing in liquid nitrogen for subsequent molecular analyses.

### 2.6 Histological observation

Hippocampal pathology was evaluated using standardized hematoxylin and eosin (H&E) histochemical protocols. Freshly dissected rat hippocampal tissues were immersion-fixed in 4% paraformaldehyde in phosphate-buffered saline (PBS; pH 7.4) for 24 h at 4 °C. Following fixation, specimens underwent graded ethanol dehydration with xylene clearing, followed by paraffin embedding. Serial 5-μm coronal sections were stained with Mayer’s hematoxylin (5 min) followed by eosin Y counterstaining (30 s) with differentiation in 0.5% acid alcohol. Histopathological analysis was performed under a microscope (NIKON, Japan) at 400 × magnification. To avoid bias, the histological slides were coded with random numbers and then assessed independently by two pathologists who were blinded to the experimental groups. Any discrepant observations were resolved through discussion until a consensus was reached.

### 2.7 Enzyme-linked immunosorbent assay (ELISA)

The concentrations of inflammatory cytokines (in serum) and brain-gut peptides (in tissue homogenates of the hypothalamus, hippocampus, brainstem, and colon) were determined by ELISA. Tissue samples were homogenized in PBS (pH 7.4) at a 1:9 (w/v) ratio and centrifuged at 3000 × g for 20 min at 4 °C. The resulting supernatant was collected after a 10-min stand and analyzed following the kit protocols, with absorbance measured at 450 nm using a microplate reader (PerkinElmer, Inc., Waltham, MA, USA). ELISA kits included: IL-1β/IL-6/TNF-*α* (70-EK301B/3–96, MultiSciences Biotech, Hangzhou, China), growth hormone (GH; ml002921, Mlbio, Shanghai, China), vasoactive intestinal peptide (VIP; H219, NJCB, Nanjing, China), substance P (SP; H218, NJCB, Nanjing, China), and cholecystokinin octapeptide (CCK8; H160, NJCB, Nanjing, China).

### 2.8 Fecal samples DNA extraction and 16S rRNA gene sequencing

Fecal microbial profiling was conducted through 16S rRNA gene sequencing (V3-V4 region). Genomic DNA was extracted using the TIANGEN Extraction Kit (DP328, TIANGEN, Beijing, China) with bead-beating lysis. DNA quality was verified by NanoDrop 2000 (A260/A280:1.8–2.0; Thermo Fisher; USA) and 1% agarose electrophoresis (150 V, 30 min). Target regions were amplified using 343F/798R primers (5’-TACGGRAGGCAGCAG-3′/5’-AGGGTATCTAATCCT-3′). Triplicate reactions were pooled, purified with AMPure XP beads (A63882, Beckman, USA), and quantified via Qubit 4.0 (Thermo Fisher, USA). Libraries were prepared per Illumina 16S Protocol and sequenced on MiSeq PE 250 (Illumina, USA). Bioinformatics analysis was performed using QIIME (V1.8.0).

### 2.9 16S rRNA bioinformatics analysis

Bioinformatic processing followed established microbiome analysis protocols. Raw paired-end reads were quality-filtered using Trimmomatic (v0.35; sliding window: 50 bp, Phred≥20). FLASH (v1.2.11; max overlap: 200 bp) merged reads with >90% identity. Chimeric sequences were removed via Vsearch (v2.4.2). High-quality tags (≥200 bp) were clustered into operational taxonomic units (OTUs) at 97% similarity. Taxonomic assignment employed the RDP classifier (80% confidence threshold) against the Greengenes database (Release 13.8) (DeSantis et al., 2006). Alpha diversity, including metrics such as Chao1, Observed Species, Shannon, and PD_Whole_Tree indices, was calculated using QIIME (v1.8.0). Group-wise differences in alpha diversity indices were assessed using the non-parametric Kruskal-Wallis test (for multiple groups) or Mann–Whitney U test (for two groups), with *post-hoc* Dunn’s test applied for multiple comparisons where appropriate. Beta diversity was calculated based on the Bray–Curtis dissimilarity metric. The overall structural differences in microbial communities between groups were statistically evaluated using complementary multivariate methods. Permutational Multivariate Analysis of Variance (PERMANOVA; adonis function, 999 permutations) was applied to test whether the group centroids were significantly distinct. To complement this and to assess the degree of separation between groups, Analysis of Similarities (ANOSIM; anosim function, 999 permutations) was performed, which provides an R statistic based on rank similarities between and within groups. Visualization of beta diversity was achieved through Principal Coordinate Analysis (PCoA) and Non-metric Multidimensional Scaling (NMDS). To identify differentially abundant taxa across groups, we employed Linear Discriminant Analysis Effect Size (LEfSe). The analysis utilized the non-parametric factorial Kruskal-Wallis test (*p* < 0.05) to detect features with significant abundance differences, followed by Linear Discriminant Analysis (LDA) to estimate the effect size of each differentially abundant feature, with a threshold of LDA score > 2.0. The functional potential of the gut microbiota was predicted from 16S rRNA data using PICRUSt2. The analysis generated KEGG Orthologs (KOs) as the primary output, which were subsequently mapped to the Evolutionary Genealogy of Genes: Nonsupervised Orthologous Groups (eggNOG) database to retrieve corresponding Clusters of Orthologous Groups (COG) functional categories and descriptions. The functional abundance profile was then constructed based on these COG annotations.

### 2.10 Correlation analysis

The Spearman correlation coefficient was used to analyze the relationships between characteristic microbiota and biochemical indicators.

### 2.11 Statistical analysis

Statistical analyses were performed using SPSS 25.0 software (IBM, Chicago, IL, USA) for all experimental data except microbiome data. Data are expressed as mean ± standard deviation (SD). The normality of data distribution was assessed using the Shapiro–Wilk test. For data meeting normality assumptions, multiple group comparisons were performed using one-way analysis of variance (ANOVA), followed by the LSD *post-hoc* test for pairwise comparisons. Differences with *p* < 0.05 were considered statistically significant.

## 3 Results

### 3.1 Hypnotic effects of abdominal massage on pentobarbital-induced sleep in rats

**Figure 1:**
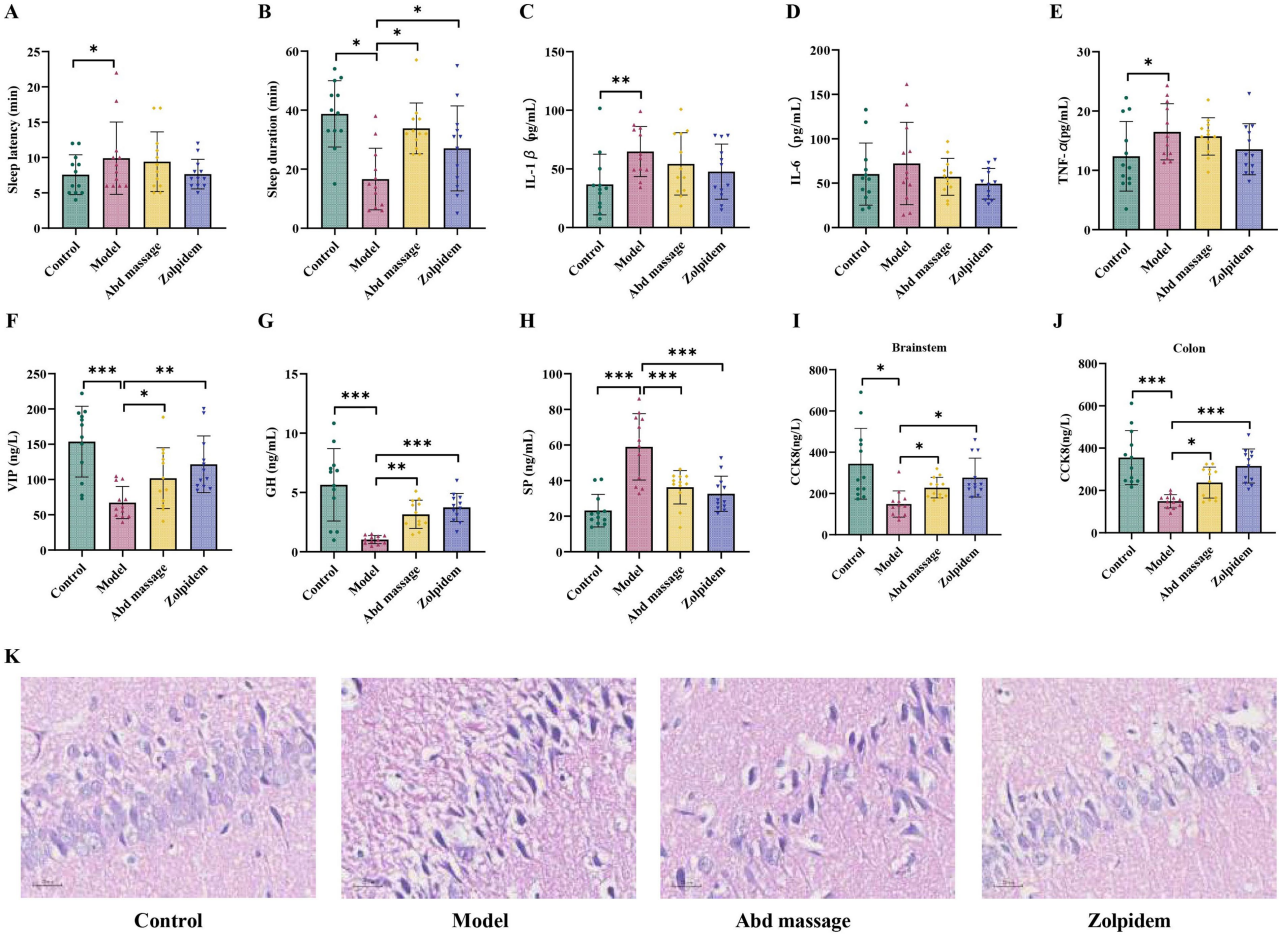
Behavioral test, ELISA, and HE staining results of rats. **(A)** Sleep latency, **(B)** sleep duration, **(C–E)** levels of inflammatory factors expression, **(F)** levels of VIP expression in the hypothalamus, **(G)** levels of GH expression in the hypothalamus, **(H)** levels of SP expression in the hippocampus, **(I)** levels of CCK8 expression in the brainstem, **(J)** levels of CCK8 expression in the colon, **(K)** histopathological evaluation of the hippocampus by H&E staining (×400). The control group displays normal neuronal architecture with intact pyramidal cells. The model group exhibits significant histopathological damage, characterized by pyramidal neuron disarray, neuronal loss, and nuclear pyknosis. These pathological changes were markedly attenuated in both the abdominal massage and Zolpidem treatment groups (***p < 0.001, **p < 0.01, *p < 0.05).

The pentobarbital sodium-induced sleep test was used to evaluate whether abdominal massage has a sleep-promoting effect. Compared to the control group, the model group exhibited prolonged sleep latency and shortened sleep duration (*p* < 0.05). Relative to the model group, both the Abd massage and Zolpidem groups demonstrated significantly increased total sleep duration (*p* < 0.05), with no intergroup differences in sleep latency (*p* > 0.05; Figures 1A,B). These findings suggest that abdominal massage exerts hypnotic sedative effects comparable to zolpidem in ameliorating sleep maintenance deficits in PCPA-induced insomnia rats.

### 3.2 Effects of abdominal massage on inflammatory factors in insomnia rats

Insomnia is often linked to inflammation and the immune system (Xiang et al., 2019). We detected the inflammation-related factors’ expression levels by ELISA. As shown in Figures 1C–E, the expression levels of inflammation-related factors IL-1β (*p* < 0.01) and TNF-*α* (*p* < 0.05) are markedly increased in rat serum after PCPA injection. In contrast, IL-6 expression showed no statistically significant difference (*p* > 0.05). These findings confirm insomnia-induced dysregulation of systemic inflammatory mediators. Both interventions demonstrated cytokine reductions without intergroup significance (*p* > 0.05).

### 3.3 Effects of abdominal massage on brain-gut peptides and hippocampal tissue morphology in insomniac rats

Quantitative analysis revealed significant brain-gut peptide dysregulation in insomnia-model rats, with hypothalamic VIP and GH levels reduced compared to controls (*p* < 0.001). The levels of CCK8 in the brainstem and colon were also significantly reduced (*p* < 0.05), while hippocampal SP showed elevation (*p* < 0.001, Figures 1F–J). Concurrently, hematoxylin–eosin staining demonstrated marked hippocampal neurodegeneration characterized by pyramidal neuron disarray, neuronal loss, and nuclear pyknosis (Figure 1I). Both therapeutic interventions effectively normalized brain-gut peptide levels and attenuated histopathological damage, though zolpidem exhibited superior amelioration of pathological damage compared to abdominal massage (Figure 1K).

### 3.4 Gut microbiota analysis

**Figure 2:**
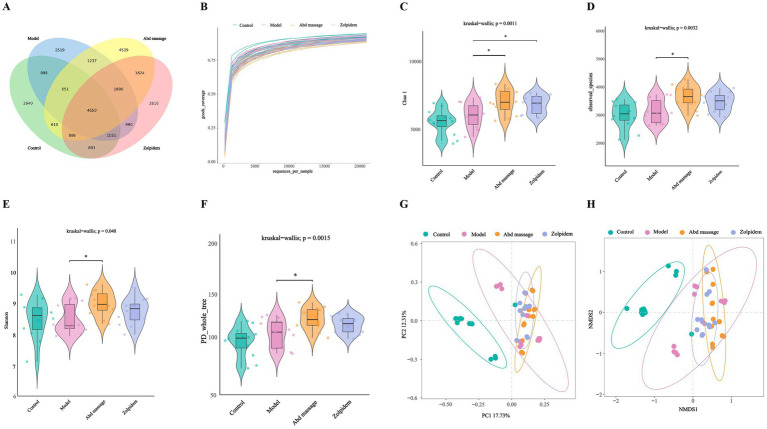
Analysis of the microbiota structure. **(A)** Venn analysis among each group, **(B)** Good’s coverage rarefaction curve, **(C–F)** Alpha diversity, **(G,H)** Beta diversity (***p < 0.001, **p < 0.01, *p < 0.05).

To investigate the potential association between the sedative-hypnotic effects of abdominal massage and gut microbiota composition, we performed 16S rRNA gene sequencing on rat fecal samples. After quality filtering, 48 samples generated 25,914–224,350 valid tags (high-quality sequences for downstream analysis), with an average read length of 410.6–417.2 bp. Microbial diversity was assessed by clustering sequences into OTUs, revealing 2,194–4,263 OTUs per sample (Supplementary material 1). Venn diagram analysis of OTUs revealed a total of 27,992 OTUs with 4,650 common species in all groups. The number of unique OTUs was 2,640, 2,519, 4,539, and 2,816 for the control, model, Abd massage, and zolpidem groups, respectively (Figure 2A). This suggests that the bacterial communities are different and change rapidly during the 14 days of treatment. The Good’s Coverage Index indicated that all groups exhibited a sample coverage exceeding 97%, affirming the high quality and reliability of the sequencing outcomes (Figure 2B).

Alpha diversity metrics were utilized to assess microbial community characteristics: species richness was quantified using Chao1 and Observed_Species indices, community diversity was evaluated via the Shannon index, and phylogenetic diversity was measured with the PD_Whole_Tree index. As shown in Figures 2C–F, no significant differences (*p* > 0.05) in alpha diversity indices were observed between the Control and Model groups. Strikingly, the Abd massage group exhibited significantly elevated alpha diversity indices compared to the Model group (*p* < 0.05), indicating that abdominal massage intervention enhanced gut microbial diversity and richness in insomnia-model rats. In contrast, zolpidem administration exerted minimal effects on microbial community diversity.

Control and Model group samples showed distinct intra-group clustering (Figures 2G,H), demonstrating substantial structural divergence between insomnia-induced and healthy rats. PERMANOVA revealed that the experimental grouping explained 27.8% of the community variation (R^2^ = 0.278, *F* = 5.658, *p* = 0.001). This finding was corroborated by ANOSIM (R = 0.550, *p* = 0.001), indicating strong separation between groups, despite the 95% confidence ellipses for the three groups partially overlapping (Supplementary material 2).

**Figure 3:**
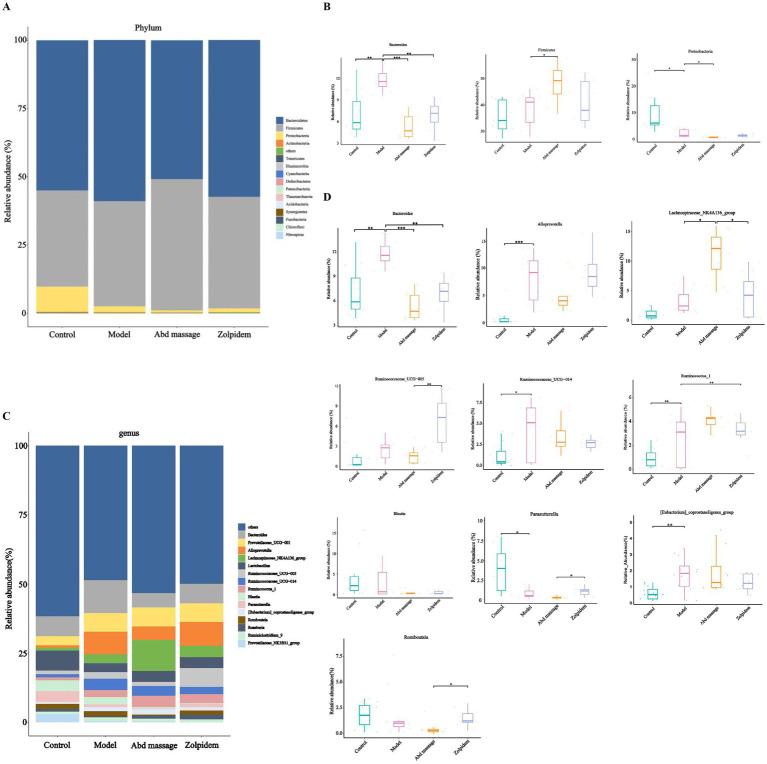
The composition of gut microbiota in rats. **(A)** Phylum-level relative abundance plot, **(B)** phylum-level dominant intestinal microbiota, **(C)** genus-level relative abundance plot, **(D)** genus-level dominant intestinal microbiota (***p < 0.001, **p < 0.01, *p < 0.05).

16S rRNA gene sequencing confirmed the relative abundance of the species at the phylum level (Figures 3A,B). Analysis of the bacterial community composition in each sample revealed that Bacteroidetes, Firmicutes, and Proteobacteria were the predominant phyla. While no significant phylum-level shifts were observed between Control and Model groups (*p* > 0.05), abdominal massage intervention (Abd massage group) significantly reduced *Bacteroidetes* and *Proteobacteria* while increasing *Firmicutes* abundance relative to the Model group. Genus-level analysis (Figures 3C,D) revealed 10 dominant taxa: *Bacteroides*, *Alloprevotella*, *Lachnospiraceae_NK4A136_group*, *Ruminococcaceae_UCG-005*, *Ruminococcaceae_UCG-014*, *Ruminococcus_1*, *Blautia*, *Parasutterella*, *[Eubacterium]_coprostanoligenes_group*, *Romboutsia*. The Model group exhibited elevated relative abundances of *Bacteroides*, *Alloprevotella*, *Ruminococcaceae_UCG-014*, *Ruminococcus_1*, and *[Eubacterium]_coprostanoligenes_group* compared to the Control group, whereas *Parasutterella* abundance was reduced. The Abd massage group demonstrated selective modulation of gut microbiota, showing increased *Lachnospiraceae_NK4A136_group* and reduced *Bacteroides*. Zolpidem treatment similarly decreased *Bacteroides*, while promoting *Ruminococcus_1* proliferation. Comparative analysis between intervention groups indicated distinct microbial signatures: Zolpidem-treated specimens showed elevated *Ruminococcaceae_UCG-005*, *Parasutterella*, *Romboutsia*, coupled with reduced *Lachnospiraceae_NK4A136_group* relative to the Abd massage group (*p* < 0.05).

**Figure 4:**
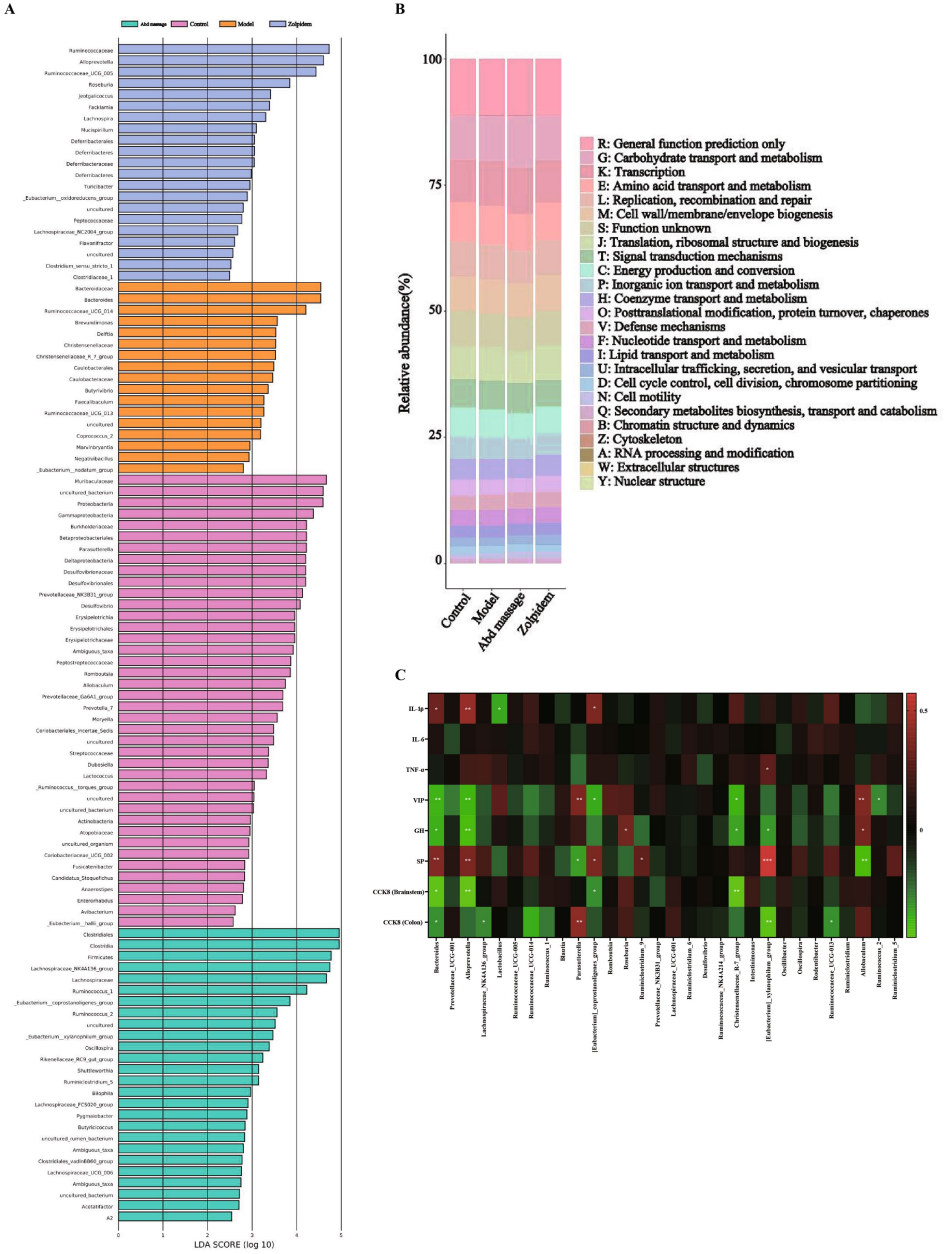
Results of LEfSe, COG functions, and Spearman correlation analysis. **(A)** LDA discriminant histogram, **(B)** COG functions, **(C)** Spearman correlation analysis (***p < 0.001, **p < 0.01, *p < 0.05).

LEfSe was implemented to detect statistically significant biomarkers across taxonomic hierarchies (LDA score >2). As shown in Figure 4A, differential abundance analysis identified 104 species with group-specific distributions: Control (40 species), Model (17 species), Abd massage (26 species), and Zolpidem (21 species). The Control group microbiota was dominated by *Muribaculaceae*, *uncultured_bacterium*, *Proteobacteria*, *c_Gammaproteobacteria*, *Deltaproteobacteria*, *Burkholderiaceae*, *o_Betaproteobacteriales*, *Desulfovibrionaceae*, *o_Desulfovibrionales*, and *Parasutterella*. The characteristic microbiota with significant differences in the model group were *Bacteroidaceae*, *Bacteroides*, *Ruminococcaceae_UCG_014*, *Brevundimonas*, *Delftia*, *Christensenellaceae*, *Christensenellaceae_R_7_group*, and *Caulobacterales*. The characteristic microbiota with significant differences in the Abd massage group were *Clostridiale*, *Clostridia*, *Firmicutes*, *Lachnospiraceae_NK4A136_group*, *Lachnospiraceae*, *Ruminococcus_1*, *Eubacterium_coprostanoligenes_group*. The characteristic microbiota with significant differences in the zolpidem group were *Ruminococcaceae*, *Alloprevotella*, *Ruminococcaceae_UCG_005*, *Roseburia*, *Jeotgalicoccus*, *Facklamia*, *Lachnospira*, *Mucispirillum*, *Deferribacterales*, etc.

In summary, the composition of the gut microbial community was significantly perturbed in rats after insomnia. However, intervention with abdominal massage and zolpidem exhibited a partial restoration of the PCPA-induced alterations in intestinal flora.

### 3.5 The PICRUSt functional prediction analysis of microbial communities

Microbial functional potential was predicted using PICRUSt2 analysis of 16S rRNA data, which generated COG annotations. Core COG functions across all groups were dominated by carbohydrate transport/metabolism, transcription, amino acid transport/metabolism, replication, recombination/repair, cell wall/membrane/envelope biogenesis, translation, ribosomal structure and biogenesis, and signal transduction mechanisms (Figure 4B). We suggested that the gut microbiota may play a positive role through the above pathways.

### 3.6 Correlation analysis between characteristic microbiota and biochemical indicators

Correlation analysis revealed significant associations between the top 30 bacterial genera and key biochemical indicators. *Bacteroides* exhibited positive correlations with IL-1β and SP, whereas it correlated negatively with VIP, GH, and CCK8 levels in both the brainstem and colon. *Alloprevotella* showed a similar pattern, being positively associated with IL-1β and SP but negatively correlated with VIP, GH, and brainstem CCK8. *Lachnospiraceae_NK4A136_group* was inversely correlated with colonic CCK8; *Lactobacillus* displayed a negative association with IL-1β. *Parasutterella* positively correlated with VIP and colonic CCK8, yet negatively with SP. *[Eubacterium]_coprostanoligenes_group* was positively associated with IL-1β and SP and negatively correlated with VIP and brainstem CCK8. *Roseburia* correlated positively with GH; *Ruminiclostridium_9* was positively associated with SP. *Christensenellaceae_R-7_group* showed negative correlations with VIP, GH, and brainstem CCK8; *[Eubacterium]_xylanophilum_group* positively correlated with TNF-*α* and SP but negatively with colonic CCK8. *Ruminococcaceae_UCG-013* was inversely associated with colonic CCK8; *Allobaculum* positively correlated with VIP and GH and negatively with SP; and *Ruminococcus_2* exhibited a negative correlation with VIP (Figure 4C).

## 4 Discussion

Insomnia is a common disease that negatively affects the health and well-being of patients. Zolpidem is a non-benzodiazepine compound and one of the most commonly used prescription drugs for insomnia, and was thus selected as a positive control. Emerging as a promising non-pharmacological intervention, abdominal massage demonstrates significant translational potential for sleep disorders. The experimental intervention was administered over the abdominal region of rats, which contains multiple acupoints. The Shenque (CV8) acupoint, anatomically centered at the umbilical region, occupies a unique position in TCM as the terminal closure site of the embryonic abdominal wall that occurred at the end of embryonic development. For this reason, it represents an excellent place to adjust the human body’s function. It is considered the focal point of the energy of life, where organs are located and from where life started through the umbilical cord connected to the mother (Zhang et al., 2019). Shenque point, characterized by structure specificity, unique position, and heat sensitivity, is not only the “reaction point” of the body’s physiological and pathological changes, but also the “stimulus point” for receiving stimulation to regulate body function. This capacity is potentiated by its microenvironment, where dense vascular networks enable sensitivity to therapeutic stimuli (Xu et al., 2021). These intrinsic characteristics substantiate CV8’s pivotal role in TCM as a functional intervention target. In addition, the abdomen contains numerous acupoints. Stimulation of the Zhongwan acupoint (CV12) modulates brain-gut peptide levels, including cholecystokinin (CCK), somatostatin (SST), and gastrin, thereby suppressing neuronal apoptosis and attenuating oxidative stress responses through coordinated neuroendocrine regulation (Yu et al., 2020). Guanyuan (CV4) acupoint regulates intestinal microbes and their metabolites (Chaoran et al., 2023). Overall, the treatment of insomnia using abdominal massage may be related to the combined actions of the immune system and the central nervous system.

The bidirectional neuroimmune communication between the central nervous system and peripheral immunity, mediated through cytokine signaling networks, has emerged as a critical modulator of sleep–wake cycle regulation (Besedovsky et al., 2019). Current evidence implicates dysregulated inflammatory responses, particularly involving interleukin (IL)-1β, IL-6, and tumor necrosis factor (TNF)-*α*, in the pathophysiology of chronic insomnia (Dolsen et al., 2019). Our experimental results align with previous studies, concentrations of IL-1β and TNF-α showed marked elevation in insomnia-model rodents compared to healthy controls (Sun et al., 2020). Contrary to expectations, abdominal massage intervention did not significantly reduce these proinflammatory mediators.

Neuropeptides are key signaling molecules in the endocrine and nervous systems that regulate many critical physiological processes. After secretion, most neuropeptides bind to G-protein-coupled receptors (GPCRs), subsequently elevating intracellular Ca^2+^ concentration. This modulates membrane excitability, transcription, and synaptogenesis, thereby regulating a broad range of behaviors, including sleep–wake cycles (Ludwig and Leng, 2006). Previous pharmacological studies indicate that vasoactive intestinal peptide (VIP) is involved in sleep regulation, particularly in promoting REM sleep. For instance, intracerebroventricular injection of VIP significantly increased REM sleep duration in rats, rabbits, and cats (Kruisbrink et al., 1987; Obal et al., 1989). Mice deficient in VIP or the VIP receptor-2 (VPAC2) exhibit significant circadian disruptions (Harmar et al., 2002; Colwell et al., 2003). Cholecystokinin (CCK) is synthesized by enteroendocrine cells in the intestinal mucosa (I cells), as well as in the brain and spinal cord of mammals. Both activated VIP and CCK8 can suppress the activity of GABAergic neurons in the hypothalamus and modulate the body’s circadian rhythm (Schwartz and Kilduff, 2015; Asim et al., 2024). The relationship between growth hormone (GH) and sleep has been studied for decades, with GH secretion being closely linked to circadian rhythms (Lyu et al., 2020). Substance P (SP) has been reported to induce sleep. For example, bilateral microinjection of SP into the ventrolateral preoptic area (VLPO) increased NREM sleep in rats, while microinjection of SP into the cerebral cortex enhanced slow-wave activity in mice (Zhang et al., 2004; Zielinski et al., 2015). In our study, significant alterations were observed in the levels of these sleep-regulating neuropeptides in both colonic and brain tissues of insomnia-model rats. Both Abd massage and Zolpidem treatment positively modulated the levels of sleep-related neuropeptides. Furthermore, examination revealed substantial mitigation of hippocampal pathology.

The gut microbiota, referred to as the second brain, can potentially influence brain homeostasis through the microbiota-gut-brain axis under both physiological and pathological conditions (Jameson et al., 2020). Pathological alterations in gut microbiota composition are frequently reported in patients with insomnia, although discrepancies exist across studies regarding specific microbial abundance (Matenchuk et al., 2020). The role of gut microbiota in TCM, as emphasized by Chu et al., is evident in our study (Chu et al., 2025). Further microbiome analyses suggest abdominal massage therapy may exert protective effects through gut microbiome restoration. Liang demonstrated that circadian rhythmicity in the abundance of *Bacteroidetes* and *Firmicutes*, the dominant phyla in mammalian gut microbiota (Liang et al., 2015). Sleep-deprived individuals exhibit elevated relative abundance of *Bacteroidetes* and *Actinobacteria*, alongside reduced levels of *Firmicutes* (Liu et al., 2019). In the present study, *Bacteroidetes* and *Firmicutes* also emerged as the dominant bacterial phyla, consistent with prior findings (Yu et al., 2022). Abdominal massage increased *Firmicutes* abundance while decreasing *Bacteroidetes* and *Proteobacteria*. *Proteobacteria*, a pathogenic genus linked to sleep disorders, produces endotoxins that drive chronic inflammation (Xiao et al., 2014; Wang et al., 2020; Agrawal et al., 2021). At the genus level, significant differences in *Bacteroides*, *Alloprevotella*, *Ruminococcaceae_UCG-014*, *Ruminococcus_1*, *[Eubacterium]_coprostanoligenes_group*, and *Parasutterella* between control and model groups suggest their potential as insomnia biomarkers. Spearman correlation analysis revealed that *Bacteroides*, *Alloprevotella*, and *[Eubacterium]_coprostanoligenes_group* were significantly positively correlated with the pro-inflammatory cytokine IL-1β, whereas *Lactobacillus* exhibited a significant negative correlation. These results suggest that *Lactobacillus*, as a beneficial bacterium, may play a key role in anti-inflammatory processes. *Lachnospiraceae_NK4A136_group*, implicated in beneficial amino acid metabolism and short-chain fatty acid production, and the higher abundance can reduce intestinal inflammation (Ma et al., 2020; Wu et al., 2021). Conversely, some studies report its negative correlation with sleep efficiency (Smith et al., 2019; Liu et al., 2023). Notably, abdominal massage increased *Lachnospiraceae_NK4A136_group* abundance, whereas zolpidem treatment reduced it. Resolution of these conflicting observations requires further investigation. Furthermore, the abundance of specific probiotics, including *Roseburia* and *Allobaculum*, showed a positive correlation with the expression levels of certain brain-gut peptides.

The LEfSe algorithm identified differentially abundant microbial taxa between experimental groups. *Bacteroides* predominated at the genus level in the model group, consistent with reports of increased abundance in PCPA-induced rodent insomnia models (Wang et al., 2024). *Bacteroidaceae* and *Ruminococcaceae_UCG-014* further characterized this group. Notably, *Ruminococcus* species serve as established markers of intestinal inflammation (Ramos and Papadakis, 2019). To validate gut microbiota rebalancing by abdominal massage therapy in insomnia models, linear discriminant analysis scores were derived from LEfSe. Liu et al. reported significant compositional, diversity, and functional disparities in insomnia patients’ microbiota versus healthy controls, identifying *Bacteroides* and *Clostridiales* as signature taxa (Liu et al., 2019). Reduced *Clostridia* and *Clostridiales* abundance correlates with impaired sleep quality (Holzhausen et al., 2024). Conversely, abdominal massage increased the abundance of *Clostridiales* and *Clostridia*, which are known to produce volatile fatty acids (VFAs) with anti-inflammatory properties. These microbial metabolites contribute to the maintenance of intestinal homeostasis (Piacentino et al., 2021; Wang et al., 2021). PICRUSt2 analysis implicates carbohydrate metabolism, amino acid metabolism, and transcriptional regulation in insomnia pathogenesis.

This study has several limitations that should be considered when interpreting the results. First, the lack of a sham massage control group prevents the definitive dissociation of the specific effects of abdominal massage from the non-specific effects of handling. Secondly, while our results provide substantial evidence implicating the MGBA in the therapeutic effects of abdominal massage, it is pertinent to consider that this may not be the sole or initial pathway. The abdominal region is a major hub for neural and circulatory networks. The mechanical pressure applied during massage could directly stimulate the vagus nerve. Furthermore, the gentle tactile stimulation itself is likely to induce a systemic relaxation response, reducing anxiety and alleviating muscular tension. These direct neurophysiological and somatosensory effects might occur independently of, or prior to, shifts in the gut microbiota, and could work in concert with the MGBA to mediate the overall hypnotic effect. Therefore, the therapeutic action of abdominal massage is likely multifaceted, engaging a complex interplay between neural, somatic, and microbial pathways.

Moreover, our study, focusing on a selected panel of brain-gut peptides, likely captures only a part of a broader neuroendocrine response. The therapeutic effects may involve other key signaling molecules, such as neurosteroids and systemic hormones, which were not measured here. Future research should incorporate sham-controlled designs and directly measure these potential pathways to establish causality and elucidate the complete therapeutic framework. In particular, employing comprehensive profiling methodologies (Chu et al., 2020; Chu et al., 2021) to simultaneously quantify a wide spectrum of neuroactive molecules would provide a more holistic view of the mechanisms involved. Despite these limitations, our work provides novel evidence linking abdominal massage to sleep improvement through gut microbiota and brain-gut peptides.

## 5 Conclusion

In summary, the results of this study demonstrate that abdominal massage ameliorates insomnia and increases sleep duration. This effect is associated with the regulation of brain-gut peptide levels and the restoration of gut microbiota diversity and structure. These findings suggest that the microbiota-gut-brain axis may be involved in the therapeutic mechanism of abdominal massage for insomnia. Future studies are warranted to establish the causal relationships underlying these observed associations.

## Data Availability

The datasets presented in this study can be found in online repositories. The names of the repository/repositories and accession number(s) can be found in the article/Supplementary material.
