# Supplementary material for: Abdominal massage modulates gut microbiota and brain-gut peptides in insomnia model rats
Source: Front Microbiol. 2025 Dec 2;16:1720248. doi: 10.3389/fmicb.2025.1720248 (PMC12705636; doi:10.3389/fmicb.2025.1720248)
Supplement: Supplementary file 1 [file Table_1.DOCX]

**Table 1 Statistical Summary of Sequences (n=48)**

| Group | Clean tags | Valid tags | Valid percent | valid min Length | valid mean Length | valid max Length | Sub sample depth | OTU counts | Total OTUs |
| --- | --- | --- | --- | --- | --- | --- | --- | --- | --- |
| Control 1 | 103672 | 88333 | 85.20% | 228 | 415.943 | 454 | 20731 | 3179 | 27992 |
| Control 2 | 81143 | 69422 | 85.56% | 228 | 416.499 | 442 | 20731 | 2750 | 27992 |
| Control 3 | 80414 | 68988 | 85.79% | 228 | 416.033 | 441 | 20731 | 3545 | 27992 |
| Control 4 | 99998 | 87138 | 87.14% | 228 | 415.800 | 441 | 20731 | 2358 | 27992 |
| Control 5 | 85018 | 69113 | 81.29% | 249 | 417.165 | 442 | 20731 | 2194 | 27992 |
| Control 6 | 69751 | 60071 | 86.12% | 236 | 415.526 | 442 | 20731 | 3382 | 27992 |
| Control 7 | 173984 | 151347 | 86.99% | 228 | 415.972 | 442 | 20731 | 3242 | 27992 |
| Control 8 | 152468 | 129683 | 85.06% | 228 | 415.465 | 442 | 20731 | 3587 | 27992 |
| Control 9 | 144182 | 123306 | 85.52% | 231 | 416.139 | 457 | 20731 | 2900 | 27992 |
| Control 10 | 87326 | 74895 | 85.76% | 229 | 416.726 | 441 | 20731 | 3032 | 27992 |
| Control 11 | 117013 | 99701 | 85.21% | 224 | 415.576 | 442 | 20731 | 3532 | 27992 |
| Control 12 | 99018 | 89100 | 89.98% | 227 | 416.013 | 449 | 20731 | 3033 | 27992 |
| Model 1 | 71318 | 60517 | 84.86% | 228 | 413.799 | 447 | 20731 | 3778 | 27992 |
| Model 2 | 76270 | 66546 | 87.25% | 227 | 414.211 | 451 | 20731 | 3569 | 27992 |
| Model 3 | 29523 | 25914 | 87.78% | 228 | 415.420 | 450 | 20731 | 2692 | 27992 |
| Model 4 | 96010 | 83278 | 86.74% | 254 | 413.046 | 440 | 20731 | 3724 | 27992 |
| Model 5 | 171606 | 149571 | 87.16% | 228 | 415.442 | 455 | 20731 | 2737 | 27992 |
| Model 6 | 151552 | 131576 | 86.82% | 228 | 413.386 | 452 | 20731 | 3781 | 27992 |
| Model 7 | 208360 | 185576 | 89.07% | 228 | 415.284 | 454 | 20731 | 2801 | 27992 |
| Model 8 | 241978 | 214853 | 88.79% | 228 | 413.724 | 444 | 20731 | 3223 | 27992 |
| Model 9 | 135915 | 121060 | 89.07% | 257 | 413.101 | 451 | 20731 | 3091 | 27992 |
| Model 10 | 57889 | 51628 | 89.18% | 224 | 413.173 | 448 | 20731 | 3095 | 27992 |
| Model 11 | 141805 | 128460 | 90.59% | 225 | 413.470 | 457 | 20731 | 2773 | 27992 |
| Model 12 | 100251 | 90492 | 90.27% | 228 | 413.805 | 453 | 20731 | 3064 | 27992 |
| Abd massage 1 | 167966 | 142447 | 84.81% | 227 | 411.414 | 442 | 20731 | 3512 | 27992 |
| Abd massage 2 | 146428 | 123904 | 84.62% | 229 | 412.859 | 440 | 20731 | 3966 | 27992 |
| Abd massage 3 | 99396 | 85254 | 85.77% | 229 | 413.298 | 438 | 20731 | 3809 | 27992 |
| Abd massage 4 | 77160 | 63467 | 82.25% | 255 | 412.638 | 440 | 20731 | 3608 | 27992 |
| Abd massage 5 | 166754 | 144591 | 86.71% | 227 | 412.336 | 443 | 20731 | 4000 | 27992 |
| Abd massage 6 | 153829 | 127898 | 83.14% | 228 | 411.138 | 440 | 20731 | 4263 | 27992 |
| Abd massage 7 | 184682 | 160198 | 86.74% | 229 | 412.011 | 455 | 20731 | 4027 | 27992 |
| Abd massage 8 | 115156 | 98720 | 85.73% | 229 | 411.773 | 441 | 20731 | 4124 | 27992 |
| Abd massage 9 | 178787 | 149887 | 83.84% | 255 | 412.846 | 442 | 20731 | 3457 | 27992 |
| Abd massage 10 | 40698 | 34629 | 85.09% | 257 | 413.786 | 441 | 20731 | 3498 | 27992 |
| Abd massage 11 | 96229 | 84764 | 88.09% | 229 | 411.441 | 441 | 20731 | 3040 | 27992 |
| Abd massage 12 | 53973 | 48416 | 89.70% | 231 | 413.013 | 450 | 20731 | 3183 | 27992 |
| Zolpidem 1 | 128613 | 113776 | 88.46% | 228 | 414.463 | 458 | 20731 | 3604 | 27992 |
| Zolpidem 2 | 112935 | 95135 | 84.24% | 228 | 410.619 | 440 | 20731 | 4000 | 27992 |
| Zolpidem 3 | 84187 | 74960 | 89.04% | 229 | 414.431 | 447 | 20731 | 3542 | 27992 |
| Zolpidem 4 | 96363 | 79950 | 82.97% | 228 | 413.270 | 441 | 20731 | 3894 | 27992 |
| Zolpidem 5 | 185872 | 161585 | 86.93% | 228 | 413.844 | 442 | 20731 | 3851 | 27992 |
| Zolpidem 6 | 135194 | 121175 | 89.63% | 223 | 414.999 | 451 | 20731 | 3327 | 27992 |
| Zolpidem 7 | 258500 | 224350 | 86.79% | 228 | 413.000 | 454 | 20731 | 3679 | 27992 |
| Zolpidem 8 | 148497 | 126830 | 85.41% | 228 | 413.173 | 452 | 20731 | 3710 | 27992 |
| Zolpidem 9 | 168363 | 144488 | 85.82% | 229 | 413.599 | 441 | 20731 | 3453 | 27992 |
| Zolpidem 10 | 113453 | 104343 | 91.97% | 228 | 414.845 | 441 | 20731 | 3014 | 27992 |
| Zolpidem 11 | 73906 | 64772 | 87.64% | 257 | 414.647 | 454 | 20731 | 3211 | 27992 |
| Zolpidem 12 | 103762 | 92953 | 89.58% | 257 | 414.258 | 442 | 20731 | 3110 | 27992 |
