# Supplementary material for: Abdominal massage modulates gut microbiota and brain-gut peptides in insomnia model rats
Source: Front Microbiol. 2025 Dec 2;16:1720248. doi: 10.3389/fmicb.2025.1720248 (PMC12705636; doi:10.3389/fmicb.2025.1720248)
Supplement: Supplementary file 2 [file Table_2.DOCX]

**Supplementary material 2**

**Tabel 2 Results of PERMANOVA based on Bray-Curtis dissimilarity**

| Source | Df | SumsOfSqs | MeanSqs | F.Model | R^2^ | Pr(>F) |
| --- | --- | --- | --- | --- | --- | --- |
| Group_factor | 3 | 2.8238 | 0.94126 | 5.658 | 0.27838 | 0.001^***^ |
| Residuals | 44 | 7.3198 | 0.16636 | / | 0.72162 | / |
| Total | 47 | 10.1435 | / | / | 1.0 | / |

Df denotes degrees of freedom; SumsOfSqs represents the total sum of squared deviations from the mean; MeanSqs (mean squares) is derived from SumsOfSqs divided by Df; F.Model indicates the F-statistic value in ANOVA testing; R^2^ quantifies the proportion of total variance attributable to grouping factors, where higher values reflect stronger between-group differentiation; Pr(>F) corresponds to the probability value, with significance levels denoted as: *** for *p* < 0.001 (highly significant); no annotation when *p* > 0.1.

**Tabel 3 Results of ANOSIM based on Bray-Curtis dissimilarity**

| Method name | ANOSIM |
| --- | --- |
| Test statistic name | R |
| Sample size | 48 |
| Number of groups | 4 |
| Test statistic | 0.55042613636363646 |
| p-value | 0.001 |
| Number of permutations | 999 |

R statistic (in ANOSIM): Measures the degree of separation between groups:R approaching 1 implies strong between-group dissimilarity relative to within-group variation; R near 0 suggests random distribution of samples across predefined groups; Negative R values (theoretically possible but biologically implausible) indicate inverted grouping patterns.
